# Supplementary material for: The Functional Convergence and Heterogeneity of Social, Episodic, and Self-Referential Thought in the Default Mode Network
Source: Cereb Cortex. 2020 Jun 23;30(11):5915–29. doi: 10.1093/cercor/bhaa166 (PMC7116230; doi:10.1093/cercor/bhaa166)
Supplement: Supplementary_Materials_20200330_bhaa166 [file supplementary_materials_20200330_bhaa166.docx]

**Supplementary Materials**


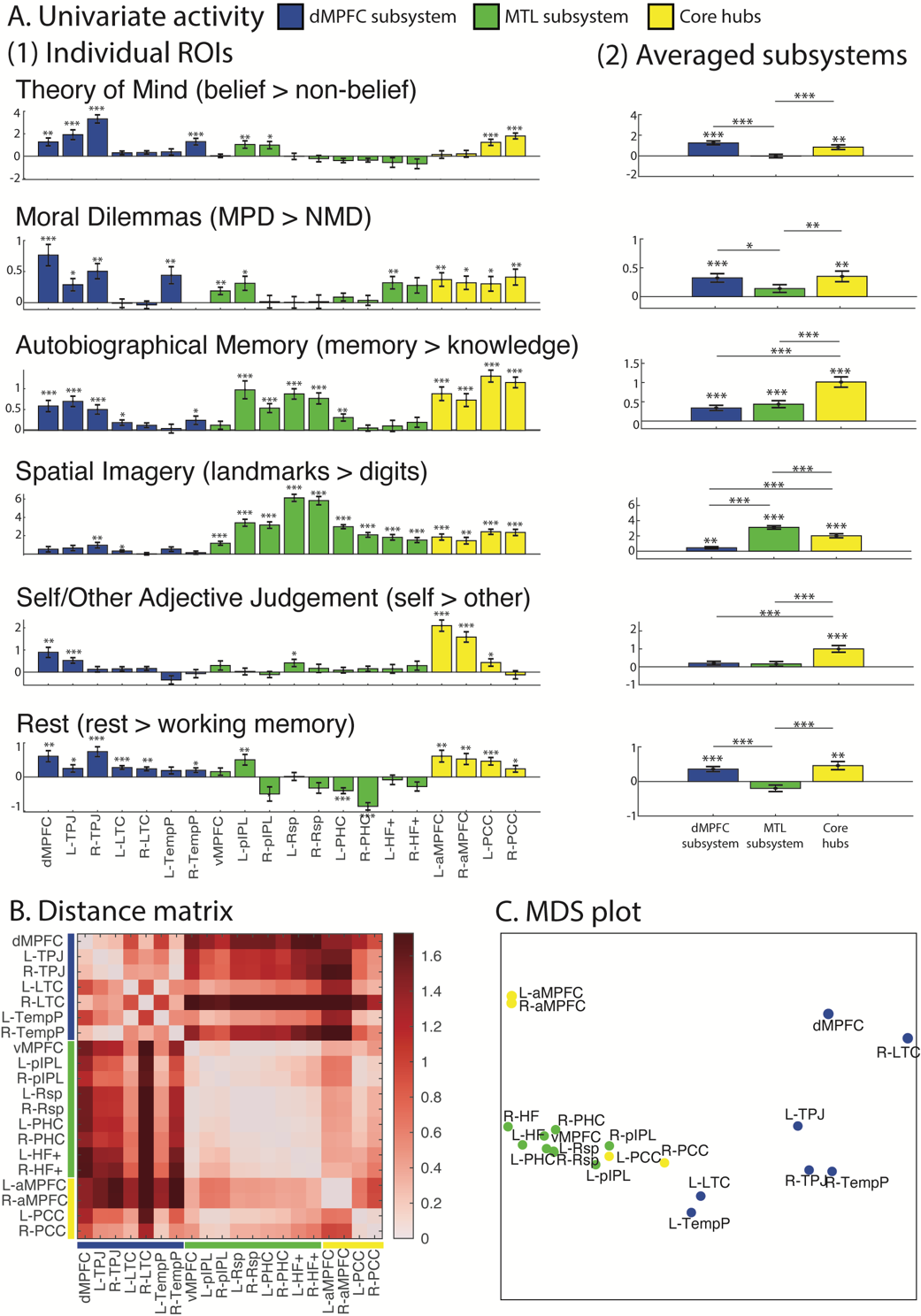


*Supplementary Figure 1. Univariate results for DMN ROIs constructed as 10 mm spheres around peak coordinates from Andrews-Hanna et al. (2010). (A) Activity (difference in beta) for each task contrast, for individual ROIs (1) and averaged over ROIs in each subsystem (2).Error bars represent standard error. t-tests against zero were conducted for each contrast. *** indicates p < 0.001, ** indicates p < 0.01, and * indicates p < 0.05 (all tests were corrected for multiple comparisons using FDR). Note that scales differ for different contrasts, and in some cases differ from scales in Figure 4. (B) Dissimilarity matrix calculated using 1 – Pearson’s r between ROIs based on their activity profile across the 6 tasks. (C) Multidimensional scaling (MDS) to visualize the dissimilarity between regions.*


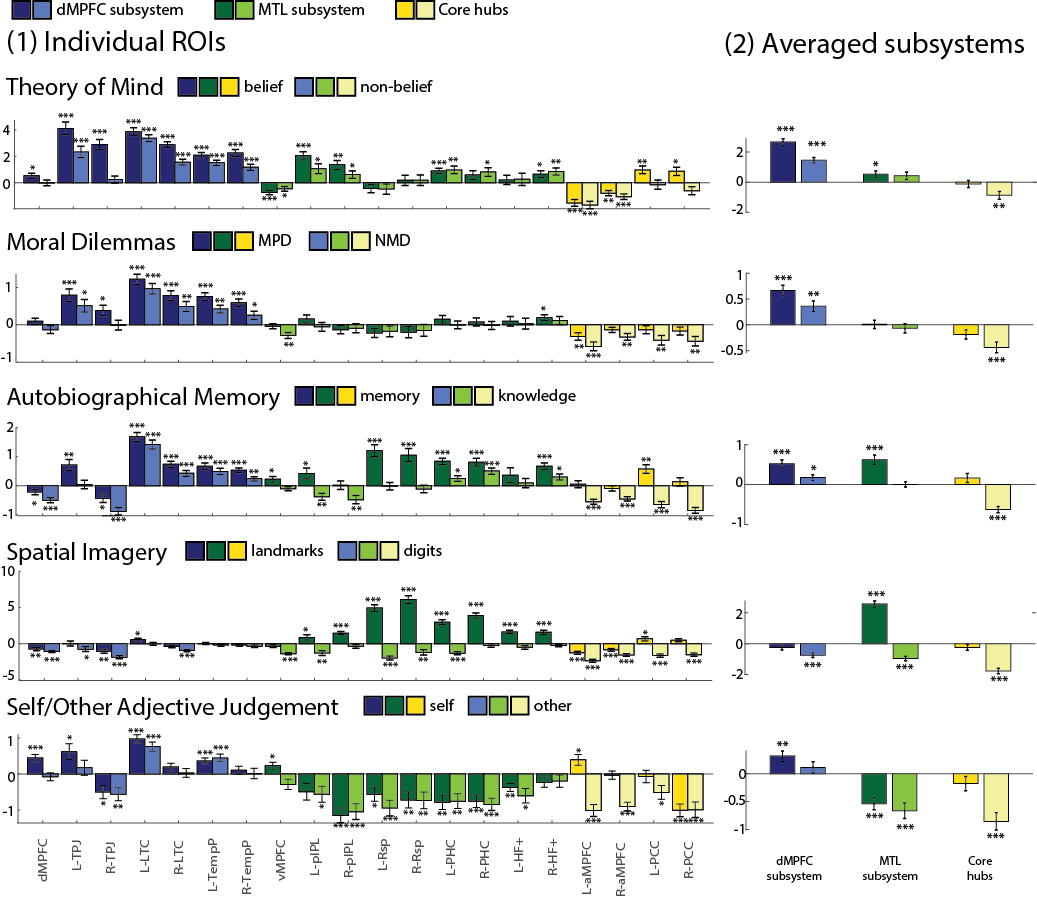


*Supplementary Figure 2. Contrasts against rest (implicit baseline). Error bars represent standard error. t-tests against zero were conducted for each contrast in each (1) ROI or (2) subnetwork. *** indicates p < 0.001, ** indicates p < 0.01, and * indicates p < 0.05 (all tests were corrected for multiple comparisons using FDR).*

*
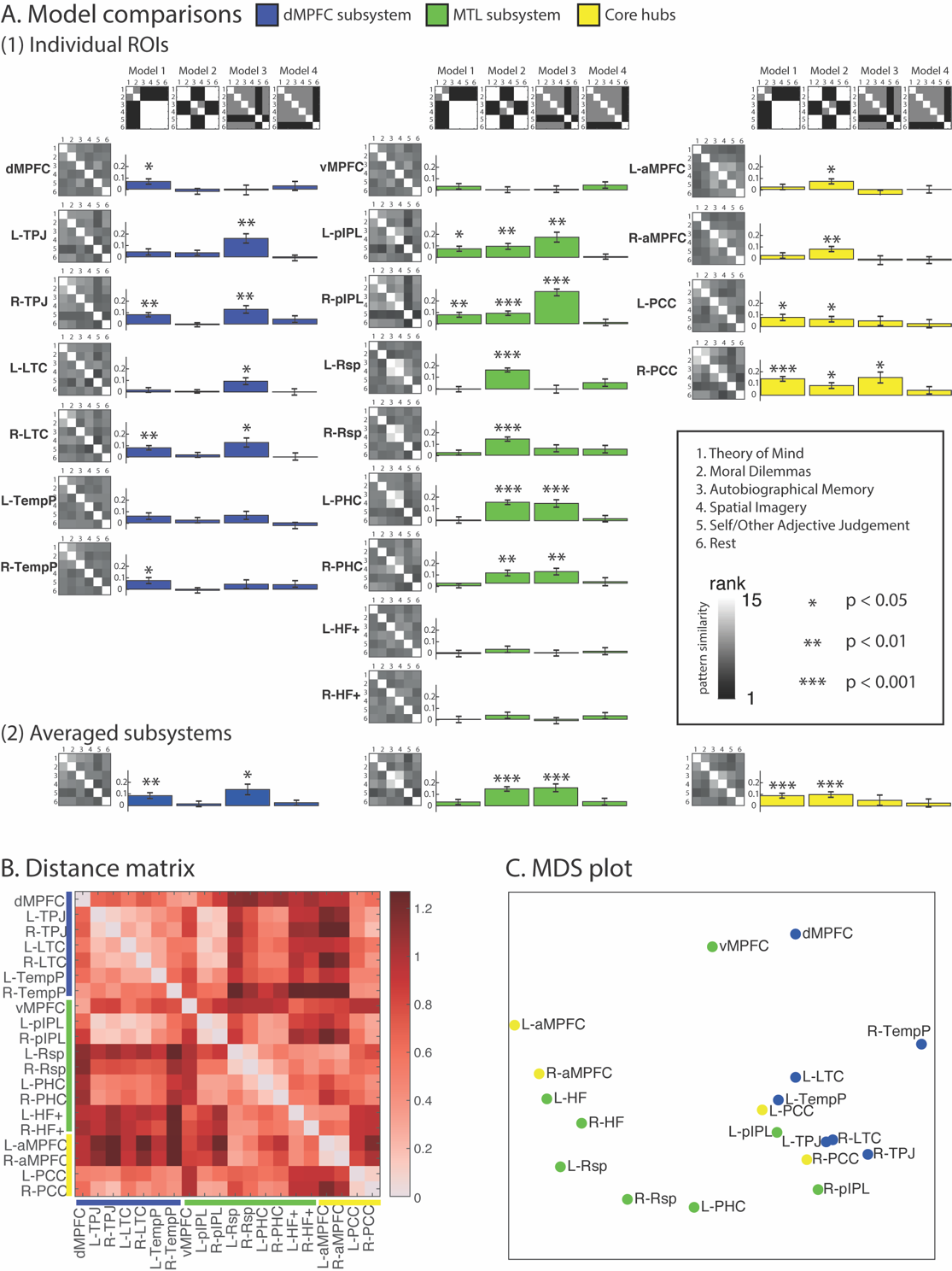
*

*Supplementary Figure 3. Multivariate results for DMN ROIs constructed as 10 mm spheres around peak coordinates from Andrews-Hanna et al. (2010). (A) (1) Similarity of multivoxel patterns for the 6 tasks, shown separately for each ROI (top to bottom in each column) in each subsystem (left, center and right columns). For each ROI, the leftmost image shows the rank-transformed similarity matrix, separately calculated for each participant and then averaged across participants. Inset shows the color scale for the data matrices. In each matrix, tasks are ordered (top to bottom and left to right) as follows: 1. theory of mind, 2. moral dilemmas, 3. autobiographical memory, 4. spatial imagery, 5. self/other, and 6. rest. To the right of each data matrix are shown correlations with four model similarity matrices (top of each column). From left to right, the models represent: high similarity for “mental state” tasks (Model 1), high similarity for “memory-based construction/simulation” tasks (Model 2), high dissimilarity of self-other to all other tasks (Model 3), high dissimilarity of rest minus task to other contrasts (Model 4). In the model matrices, white indicates empty cells not used in the comparisons, gray indicates 1s (similar), and black indicates 0s (dissimilar). Bar graphs indicate the Kendall’s tau-a correlation between each participant’s empirical and model similarity matrices tested against zero (corrected for multiple comparisons at FDR < 0.05; *** indicates p < 0.001, ** indicates p < 0.01, and * indicates p < 0.05). (2) Equivalent results averaged across ROIs within each subnetwork. (B) Dissimilarity matrix calculated using 1 – Pearson’s correlation between ROIs based on their correlation profiles across 15 task pairs. (C) Multidimensional scaling (MDS) to visualize the dissimilarity between regions.*
